# Diffuse alveolar hemorrhage as a presentation of severe anicteric leptospirosis

**DOI:** 10.1590/0037-8682-0582-2023

**Published:** 2024-02-05

**Authors:** Andrés Eduardo Prieto Torres, Sergio Andrés Bolívar-Lozano, Álvaro A. Faccini-Martínez

**Affiliations:** 1 Hospital Militar Central, Servicio de Medicina Interna, Bogotá D.C, Colombia.; 2 Hospital Militar Central, Servicio de Infectología, Bogotá D.C, Colombia.; 3 Universidad Militar Nueva Granada, Facultad de Medicina, Bogotá D.C, Colombia.; 4 Servicios y Asesorías en Infectología - SAI, Bogotá, D.C, Colombia.

**FIGURE 1:**
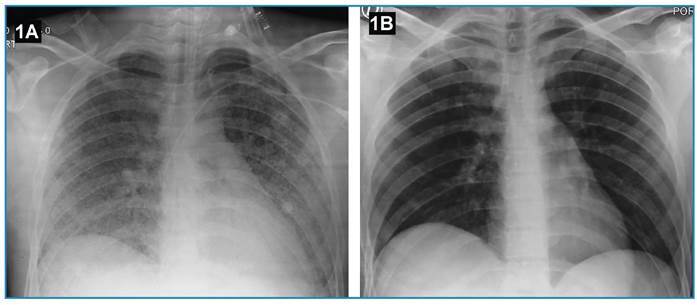
Chest radiograph showing mixed opacities distributed in the parenchyma of all four quadrants of both lungs, without consolidations **(A)**; and complete resolution of the lung infiltrates following treatment **(B)**.

**FIGURE 2:**
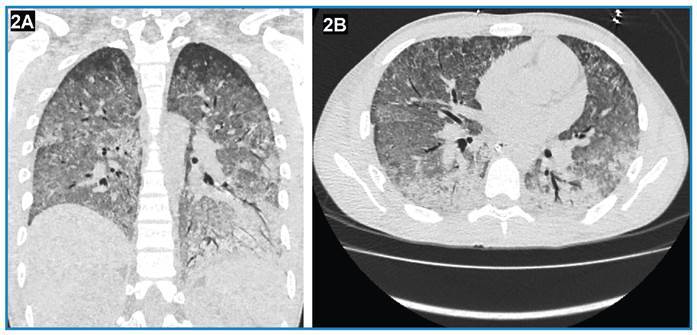
High-resolution chest computed tomography (coronal **(A)** and transverse **(B)** planes) showing alteration in the attenuation coefficients of the lung parenchyma due to diffuse ground-glass attenuation of the parenchyma of both lungs, with thickening of the interlobular and intralobular septa and bilateral consolidation in the lower lobes, primarily in the basal segments.

An 18-year-old Colombian male soldier was referred to our institution for a febrile illness that had progressively worsened over 8 days. His symptoms included fever, general malaise, cough, and hemoptysis, which progressed to respiratory distress, necessitating orotracheal intubation. Chest radiography revealed diffuse bilateral mixed opacities (Figure 1A). A complete blood count showed leukocytosis and neutrophilia, and blood biochemistry showed elevated creatine and phosphokinase levels. Both a rapid IgM test and an IgM ELISA were positive for *Leptospira*. High-resolution chest computed tomography (Figure 2A and 2B ) revealed a classic pattern of diffuse alveolar hemorrhage, which was subsequently confirmed by bronchoscopy and bronchoalveolar lavage. We initiated ceftriaxone and methylprednisolone pulse therapy at a dose of 1000 mg/day for 3 days, followed by 1 mg/kg/day for 7 days. After treatment, the patient’s clinical condition rapidly improved, enabling successful weaning from mechanical ventilation (Figure 1B). A serum sample collected on day 8 of illness was retrospectively tested using a microscopic agglutination test, which yielded a positive result for *Leptospira kirschneri* serogroup Cynopteri with a dilution of 1:3,200. 

Diffuse alveolar hemorrhage is a rare but potentially life-threatening presentation of human leptospirosis that usually occurs during the icteric phase of the illness^1^ ^,^ ^2^. However, it can occur during the anicteric phase in a small percentage of patients, posing a clinical diagnostic challenge, particularly in critically ill patients^1^ ^,^ ^2^. Although randomized clinical trials are lacking, the use of steroids for the treatment of leptospirosis with diffuse alveolar hemorrhage leads to favorable outcomes and is generally safe^3^.
